# Full Open Access Journals Have Increased Impact Factors

**DOI:** 10.3390/molecules14062254

**Published:** 2009-06-22

**Authors:** Shu-Kun Lin

**Affiliations:** Molecular Diversity Preservation International (MDPI), Matthaeusstrasse 11, CH-4057 Basel, Switzerland; Tel. +41-79 322 3379; Fax: +41-61 302 8918; E-mail: lin@mdpi.org; http://www.mdpi.org/lin/

**Figure 1:**
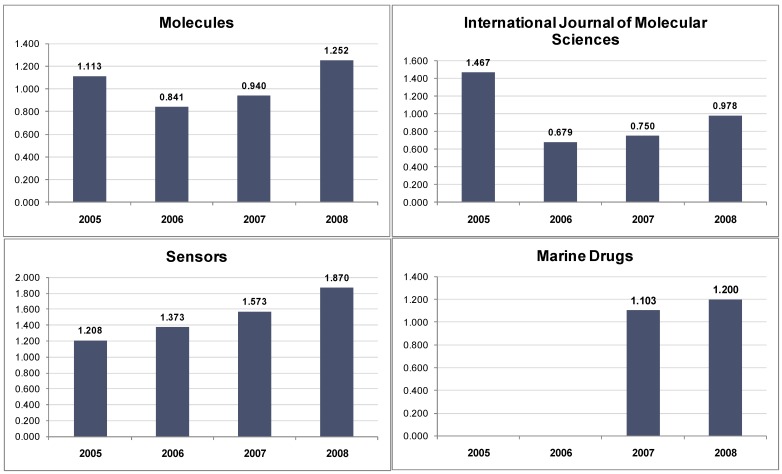
Impact factors of four MDPI journals (adapted from the Journal Citation Report (JCR), Edition 2008, Copyright 2009 by Thomson Reuters).

We are pleased to report the increase of the impact factors of MDPI journals during 2007 and 2008 (see Figure 1). In 2005 and part of 2006, the use of a two tier publication system, whereby we offered full Open Access publication to those authors willing to contribute financially to support this option, while providing the alternative choice of free publication without Open Access for those authors who preferred not to pay [1,2], resulted in the obviously decreased impact factors seen in 2006 [3,4]. In early 2007, a full Open Access publishing policy was instituted and we can now begin to clearly see the effect of the full Open Access policy in the steady recovery of the impact factors of the affected journals. 

On the other hand, the two tier publication system (Open Access and non-Open Access) was only briefly applied to the journal *Sensors* and we thus see a continuous increase of the impact factor in recent years. The 2008 impact factor of the journal *Sensors* is 1.870. 

We also observed an interesting phenomenon: two *Molecules* papers were retracted because they had also been published elsewhere. Nevertheless, these withdrawn papers were cited elsewhere, while the non-Open Access papers were not. We have now added a watermark to the original PDF files of withdrawn papers, clearly identifying their status, in order to prevent further citation of such papers [5]. 

Since October 2008, all MDPI journals have been published at the new server www.mdpi.com. Just recently we published the 5000th paper in a MDPI journal and *Molecules* alone has published over 2000 papers. As we wrote in our editorial [6], the year 2009 will be a more successful year for the journal *Molecules* and for all the other MDPI journals, including several newly launched journals.
